# Biomimetic Silicone Surfaces for Antibacterial Applications

**DOI:** 10.3390/polym17020213

**Published:** 2025-01-16

**Authors:** Marie Barshutina, Dmitry Yakubovsky, Aleksey Arsenin, Valentyn Volkov, Sergey Barshutin, Anastasiya Vladimirova, Andrei Baymiev

**Affiliations:** 1 Moscow Center for Advanced Studies, Moscow 123592, Russia; 2 Emerging Technologies Research Center, XPANCEO, Dubai P.O. Box 393047, United Arab Emirates; 3 Institute of Power Engineering, Instrument Engineering, and Electronics, Tambov State Technical University, Tambov 392000, Russia; 4 Institute of Biochemistry and Genetics, Subdivision of the Ufa Federal Research Center of the Russian Academy of Sciences, Ufa 450054, Russia

**Keywords:** silicone interfaces, antibacterial effect, flower petals, biomimetic, patterned surfaces, fractal analysis

## Abstract

Biomimetic patterning emerges as a promising antibiotic-free approach to protect medical devices from bacterial adhesion and biofilm formation. The main advantage of this approach lies in its simplicity and scalability for industrial applications. In this study, we employ it to produce antibacterial coatings based on silicone materials, widely used in the healthcare industry. In doing so, we patterned silicone substrates with a topography of various flower petals (rose, chamomile, pansy, and magnolia) and studied the relationship between the antibacterial properties of the obtained biomimetic substrates and their surface topography. To study the surface topography of biomimetic surfaces, we used the fractal analysis of their SEM images. In particular, as a measure of surface complexity and heterogeneity, we used the values of the developed interfacial area ratio (*Sdr*) and lacunarity coefficient (β). In the result, we found that the bacterial area coverage of biomimetic substrates decreased exponentially with the increase in their surface complexity and heterogeneity, and prominent antibacterial properties were observed at β > 1.6 and *Sdr* > 50. The results of this study can be used to identify biomimetic materials with superior antibacterial properties and produce efficient antibacterial silicone coatings for biomedical and healthcare applications.

## 1. Introduction

Polydimethylsiloxane (PDMS) is a high-performance polymer, with unique physical and chemical properties, such as flexibility, thermal stability, oxidation resistance, inertness to body fluids, non-toxicity, and biocompatibility. Due to these unique properties, PDMS is widely used for the fabrication of various biomedical devices (e.g., ureteral stents, catheters, endotracheal tubes, contact lenses, voice prosthesis, aesthetic implants, etc.). However, a major disadvantage of PDMS is its surface chemistry favorable for the adhesion of bacteria, which can cause severe infections, device failure, or even death [1,2]. The current strategies to prevent bacterial infections related to medical devices are usually based on the use of prophylactic or therapeutic antibiotics. However, their effectiveness is gradually decreasing due to the rapid emergence of antibiotic-resistant bacteria. As a consequence, research teams all over the world focused their efforts on the development of antibiotic-free strategies to protect medical devices from bacterial adhesion and biofilm formation. Some of the most promising antibiotic-free strategies proposed so far are the use of nanomaterials [3,4,5] and surface patterning with nano- or micro-structures [6,7,8,9]. A number of research groups [10,11,12,13] have reported that nanoscale structures smaller than bacterial cells (<1 μm) can prevent bacteria from attachment or even kill them on contact by piercing the cell walls. On the other hand, microscale structures close in size to bacteria (~1–10 μm) may also be effective for antibacterial applications, since they lead to the alignment of bacterial cells and inhibit biofilm formation by blocking cell–cell communications [14,15,16,17,18].

In the last decade, the nano/micro-patterning strategy has been applied for the fabrication of silicone surfaces with antibacterial and anti-biofouling properties. In particular, Hechmann et al. [19] and Barshutina et al. [20] produced silicone substrates patterned with pillars of nano-scale sizes and demonstrated their effectiveness against *Escherichia coli* (*E. coli*) and *Staphylococcus aureus* (*S. aureus*) bacteria. Additionally, several research teams [21,22,23,24] have fabricated silicone surfaces with micro-scale topography and reported their anti-fouling properties toward Gram-positive and Gram-negative bacteria, which is highly promising for various biomedical applications.

To produce silicone surfaces patterned with nano- or micro-structures, various types of lithography [19,20,21,22], etching [19,20], and laser machining techniques [23,24] are generally applied. However, the widespread deployment of these methods for the patterning of biomedical devices is highly limited due to the complexity and high cost of manufacturing processes. To overcome this issue, a biomimetic approach has been recently proposed as an alternative to conventional patterning techniques. A number of researchers have demonstrated that silicone materials with antibacterial properties can be fabricated in a simple and cost-effective way by replicating the topography of natural surfaces, such as the skin of marine creatures, plant leaves, etc. [25,26]. For instance, Lin et al. [25] produced micro-patterned silicone membranes by a replica molding technique using dried shark skin as a template. The obtained silicone membranes exhibited a pronounced antibacterial effect and great potential for wound-healing applications. Zhao et al. [26] applied the same technique and *Laminaria japonica* leaves as a template to fabricate micro-patterned silicone films with anti-fouling properties. The anti-fouling performance of the obtained films was evaluated by using *E. coli* suspensions, and their anti-fouling efficiency was about 96.2%. For comparison purposes, all the above studies on nano- and micro-patterned silicone coatings with antibacterial properties are summarized in Table S1 in Supplementary Materials.

Overall, the biomimetic strategy has proven itself as highly promising for the fabrication of silicone surfaces with antibacterial and anti-fouling properties. However, further research in this field is highly needed to reveal the antibacterial mechanisms of biomimetic surfaces in more detail, compare the antibacterial efficiency of biomimetic surfaces inspired by various natural objects, and find a way to predict their antibacterial properties through topography analysis.

The present work is devoted to the antibacterial and fractal image analysis of silicone surfaces patterned with a hierarchical topography of flower petals. For the experiments, we chose petals from widely available plant species, such as rose, chamomile, pansy, and magnolia. A thorough study of the correlation between a flower petal topography and its antibacterial efficiency allowed us to develop an algorithm predicting the antibacterial properties of biomimetic surfaces based on the fractal analysis of their SEM images. Additionally, we studied the antibacterial mechanism of various biomimetic silicone surfaces and revealed the main topographical factors contributing to their antibacterial activity. The obtained results can be used to improve and scale-up the fabrication of antibacterial silicone coatings, which is highly promising for various biomedical and healthcare applications.

## 2. Materials and Methods

### 2.1. Fabrication of Biomimetic Surfaces

**Figure 1:**
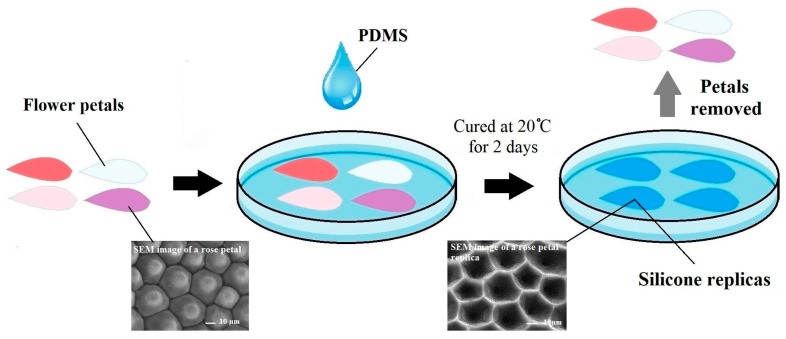
Schematic of the fabrication process to obtain silicone replicas of flower petals.

The fabrication of biomimetic silicone substrates was performed by a replica molding (or soft lithography) technique. A silicone elastomer for flower petal replication was prepared using commercially available Sylgard-184 (Dow Corning, Midland, MI, USA) with a base/curing agent mixing ratio of 10:1. Fresh roses (*Rose Hybrid Tea*), pansies (*Viola tricolor var. Hortensis*), daisies (*Bellis perennis*), and magnolias (*Magnolia liliiflora*) were taken from the local garden. Several pieces (~1 cm^2^) of petals from each flower were fixed in a Petri dish with adhesive tape. The silicone elastomer was poured over the petal pieces in the Petri dish and cured at standard room temperature (20 °C) for 2 days. After curing, the silicone replicas were gently peeled off and washed with distilled water. A schematic representation of the fabrication technique is presented in Figure 1.

### 2.2. Characterization of Biomimetic Surfaces

The silicone replicas of flower petal surfaces were visualized by SEM (SEM, JSM7001F, JEOL, Tokyo, Japan), which was operated at an acceleration voltage of 30 kV and current of 67 µA. To avoid the influence of the surface electrical charging of the silicone surface during SEM measurements, a thin titanium layer was preliminarily deposited on the silicone replicas. Microscope images (1600 × 1200 pixels) of samples were also captured with an optical microscope (Nikon LV150L, Tokyo, Japan) equipped with a color digital camera DS-Fi3.

### 2.3. Antibacterial Properties of Biomimetic Surfaces

A bacterial cell line of *E. coli* (ATCC 25922, Thermo Fisher Scientific, Waltham, MA, USA) labeled with the green fluorescent protein TurboRFP (excitation/emission max = 553/574 nm) was used as a model to evaluate the antibacterial ability of biomimetic substrates [27]. Notably, a flat silicone substrate was used as a reference specimen. Six samples of each substrate with a size of 1 × 1 cm^2^ were sterilized using an ultraviolet lamp for 30 min, placed into 6-well non-tissue culture plates filled with tryptic soy broth (TSB, 0.03 g/mL, 1 × 10^7^ bacteria/mL), and incubated at 37 °C for 48 h. The medium was changed with 1 mL of sterile and fresh TSB after 24 h. Afterward, the samples were rinsed twice with phosphate-buffered saline to remove any non-adherent transient bacteria. Bacteria attached to pristine silicone and biomimetic silicone surfaces were visualized using a Biozero BZ-8100 Fluorescence Microscope (Keyence, Osaka, Japan) with excitation and emission filters of BP 546/12 and LP 590, respectively. Quantitative analysis of the fluorescent images was performed using ImageJ software (ImageJ version 1.54i, NIH, Bethesda, MD, USA). At least 6 representative images at a magnification of 20× were used to calculate the E. coli area coverage (EAC). These statistical analyses were conducted using a threshold of *p* < 0.05. Three independent experiments were performed for each surface type and their results were averaged.

### 2.4. Topography Analysis

#### 2.4.1. Lacunarity Analysis

Lacunarity analysis is a tool used for the fractal analysis of surface topography. It can be applied to characterize data and geometric patterns in various areas such as ecology, physics, medical imaging, urban spatial analysis, etc. [28,29,30]. The lacunarity coefficient β evaluates the size distribution of gaps on the surface of the fractal object and provides a quantitative measure of its heterogeneity. A more heterogeneous distribution of gap sizes causes higher values of the β coefficient. It should be noted that homogeneous surfaces at a large scale can be heterogeneous at a smaller scale, and vice versa. The difference in the lacunarity coefficient at a particular scale can be used to indicate the difference in homogeneity on that scale.

Several efficient algorithms have been proposed so far to calculate the coefficient of lacunarity [31,32,33,34,35,36]. One of them, called the sliding box algorithm [35], was applied in this study to determine the lacunarity of biomimetic surface images. Prior to lacunarity analysis, grayscale SEM images of biomimetic surfaces were binarized (i.e., each pixel was assigned with a value of 1 or 0 depending on whether its grayscale intensity was above or below the median value, respectively). Then, the sliding box algorithm was implemented in the following steps: (1) The sliding box of *r* × *r* size was placed at the top-left corner of an image of *N* × *N* size. (2) The sliding box was moved through the binary image one pixel at a time and the “mass” *M* of each box was calculated as the number of pixels occupied with “1”. (3) The number of boxes with “mass” *M* not equal to 0 was denoted as *n* (*M*, *r*), and the total number of boxes was defined as *T* (*r*) = (*N* − *r* + 1)^2^. (4) The probability distribution of occupied boxes was determined as *Q* (*M*, *r*) = *n* (*M*, *r*)/*T* (*r*). (5) The lacunarity coefficient for the box of size *r* × *r* was obtained as: β = *K*_2_/*K*_1_^2^, where *K*_1_ = Σ (*M* × *Q* (*M*, *r*)) and *K*_2_ = Σ (*M*^2^ × *Q* (*M*, *r*)) are the first and second moments of the distribution *Q (M*, *r)*, respectively. (6) Finally, the dependence of the lacunarity coefficient on the sliding box size *r*, or so-called “lacunarity spectrum”, was plotted for *r* values in the range from 1 to *N*. In this study, the sliding box algorithm was implemented in the MathLab programming environment (MathLab version R2013a, MathWorks, Natick, MA, USA).

#### 2.4.2. Analysis of Developed Interfacial Area Ratio

The developed interfacial area ratio (*Sdr*) is used as a measure of surface complexity, which can be calculated as the ratio of the real surface area to the projected area defined by the scan length [37]. According to recent studies [38,39], there is a strong correlation between the *Sdr* of patterned surfaces and their mechanical, optical, thermal, and biological properties. In this study, we used the *Sdr* parameter to predict the antibacterial and anti-fouling properties of micro-patterned biomimetic surfaces.

To obtain the values of the *Sdr* for the examined biomimetic substrates, we used fractal analysis of their SEM images, which includes the following steps: (1) The grayscale SEM images of biomimetic substrates were expanded into a linear matrix of *n* elements, where each matrix element of size *a* × *a* corresponds to a pixel of the image. (2) The obtained matrix was processed by a wavelet transform function in order to remove artifacts and noise from the image data [40]. (3) The color intensity of each matrix element was converted to heights using the values of the highest points of microstructures in SEM images of sample sections (see Figure S1 in Supplementary Materials). (4) The real surface area of biomimetic substrates was calculated as *l* = Σ (*a*^2^ + (*h_i_* − *h_i−1_*)^2^)^1/2^, where *h_i_* is the height of the *i*-th matrix element and *h_i-1_* is the height of the (*i* − 1)-th element (for *i* ranging from 1 to *n*). (5) Finally, the *Sdr* of SEM images was estimated as: *Sdr* = *l*/(*n* × *a*). The proposed algorithm was implemented in the MathLab programming environment.

## 3. Results and Discussion

### 3.1. Characterization of Biomimetic Surfaces

**Figure 2:**
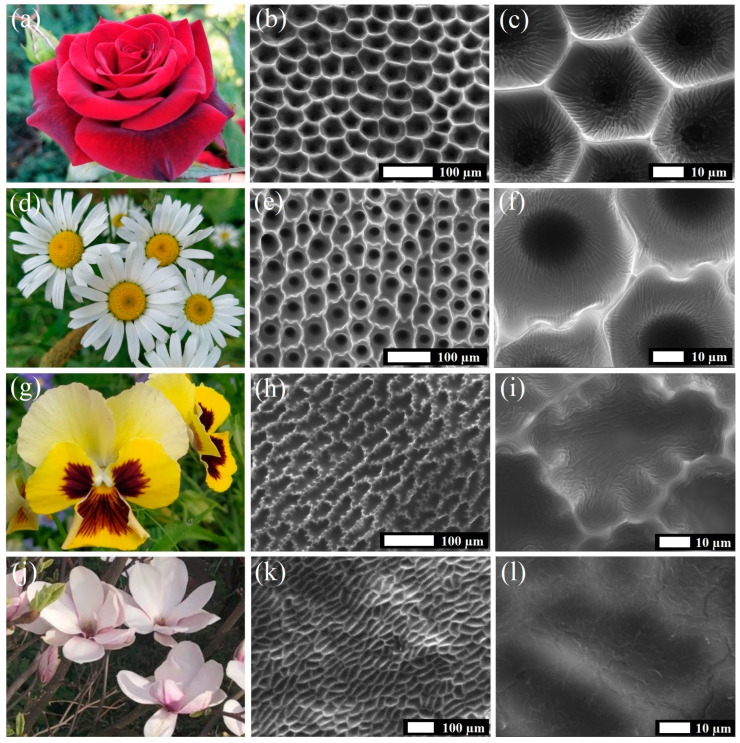
SEM images illustrating the silicone replicas of rose, chamomile, pansy, and magnolia petals: (**a**,**d**,**g**,**j**) Photograph of a flower; (**b**,**e**,**h**,**k**) silicone replica of a flower petal with a 100 µm scale bar; (**c**,**f**,**i**,**l**) silicone replica of a flower petal with a 10 µm scale bar.

SEM imaging of the flower petal replicas (Figure 2) revealed the presence of periodic arrays of cavity-shaped hierarchical structures on their surfaces. In particular, the silicone replicas of rose petals (Figure 2b) were covered with an array of honeycomb-like cavities of about 25–35 µm in diameter. The magnified SEM image of these cavities (Figure 2c) demonstrates the presence of submicron foldings of 700–900 nm in width at their walls and bottom. Similar, but more tortuous, micro-scale cavities of about 35–45 µm in diameter were observed on the surface of chamomile petal replicas (Figure 2e,f). The walls of these cavities were also covered with sub-micron foldings, but their relief was much less pronounced. Another type of periodic cavity can be observed on the surface of pansy and magnolia petal replicas. In particular, the surface of pansy petal replicas was formed with elongated tortuous cavities of about 35–45 µm in width and 60–90 µm in length (Figure 2h,i). Similar, but less tortuous, oblong cavities could be seen on the surface of magnolia petal replicas (Figure 2k,l). The width and length of these cavities were 25–35 µm and 60–80 µm, respectively. Additionally, the inner surface of cavities covering pansy and magnolia petal replicas was formed with low relief foldings of sub-micron scale (600–900 nm). Overall, based on the visual assessment of SEM images, we can say that flower petal replicas have highly complex and heterogeneous surfaces. However, to quantify and compare the degree of their complexity and heterogeneity, we performed fractal analysis of the SEM images, the results of which are presented below.

### 3.2. Fractal Analysis of SEM Images

#### 3.2.1. Lacunarity Analysis

**Figure 3:**
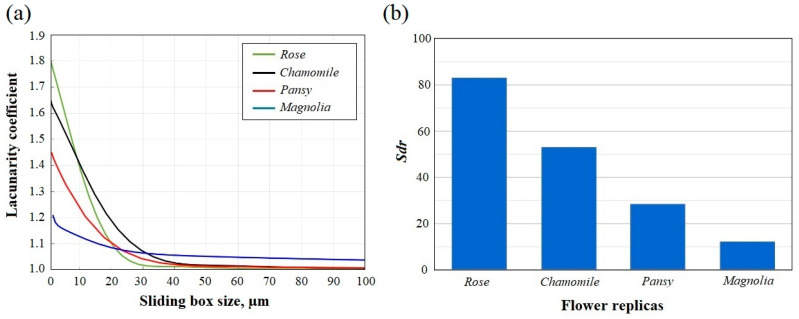
Fractal analysis of SEM image topography of flower petal replicas: (**a**) lacunarity analysis; (**b**) roughness ratio analysis. The relative standard deviation (RSD) of all measurements was less than 5%.

To measure the surface heterogeneity of flower petal replicas at various scales, we performed lacunarity analysis of their SEM images [29]. The results of lacunarity analysis are presented in Figure 3a.

Significant differences in lacunarity between flower petal replicas can be observed at the scales of *r* < 20 µm. At these scales, the maximum difference (up to 60%) appeared between rose and magnolia petal replicas, indicating a great difference in the heterogeneity of their surfaces. The lacunarities of pansy and chamomile petal replicas were also superior to that of the magnolia one, and the differences in their lacunarity reached 24% and 43%, respectively. Thus, at small scales, the surface heterogeneity increased rapidly in the row: magnolia, pansy, chamomile, and rose petal replicas. At larger scales (*r* > 40 µm), all flower replicas demonstrated a low level of lacunarity corresponding to a relatively homogenous structure of their surfaces. Additionally, the lacunarity graphs of all substrates had more or less pronounced inflections at scales of 20 µm < *r* < 40 µm, coinciding with the micro-scale periodicity of their biomimetic structures (Figure 2). Hence, we can conclude that the biomimetic surfaces were highly homogeneous at scales above the size of their periodic structures, and heterogeneous at smaller scales. Considering that a high level of surface heterogeneity at small scales (comparable with bacteria sizes) promoted the antibacterial efficiency of patterned substrates [41], we can expect pronounced antibacterial properties in biomimetic surfaces with a high level of lacunarity, such as rose and chamomile petal replicas.

#### 3.2.2. Analysis of Developed Interfacial Area Ratio

To measure the surface complexity of flower petal replicas, we used the *Sdr* parameter, which is determined as the ratio of the real surface area to the projected one. The *Sdr* values for rose, chamomile, pansy, and magnolia replicas are presented in Figure 3b. According to the obtained bar chart, the degree of surface complexity decreased significantly in the row: rose, chamomile, pansy, and magnolia petal replicas. For instance, the *Sdr* values of chamomile and rose replicas exceeded the *Sdr* value of magnolia replicas by more than six and four times, respectively. According to recent literature [8], the increased level of surface complexity at nano- and micro-scale levels contributes to the appearance of antibacterial properties. Thus, rose and chamomile replicas characterized by large values of developed interfacial area ratio had strong prerequisites for the manifestation of antibacterial activity.

### 3.3. Analysis of Antibacterial Properties

**Figure 4:**
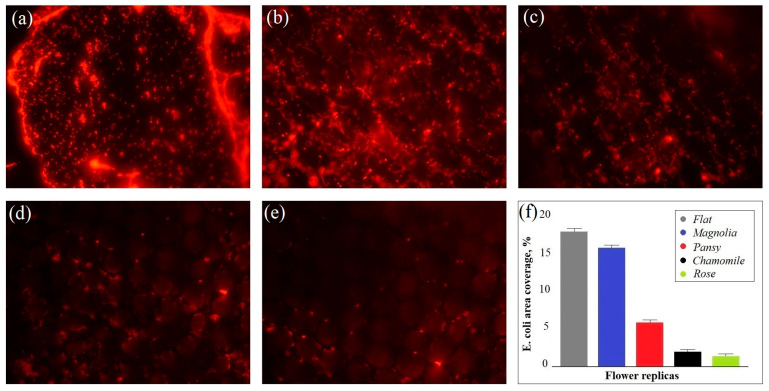
The fluorescent images of biomimetic and flat substrates populated with bacteria (at 20× magnification): (**a**) flat; (**b**) magnolia replica; (**c**) pansy replica; (**d**) chamomile replica; (**e**) rose replica; (**f**) quantitative analysis.

Further experimental studies with an *E. coli* culture have confirmed that biomimetic silicone substrates can vary greatly in their antibacterial properties depending on the degree of surface complexity and heterogeneity. In these studies, we used a flat silicone substrate with a highly uniform and homogeneous surface as a reference sample. The fluorescent images of biomimetic and flat substrates after 48 h of incubation in the *E. coli* media are presented in Figure 4a–e. The results of their quantitative analysis are shown in Figure 4f.

**Figure 5:**
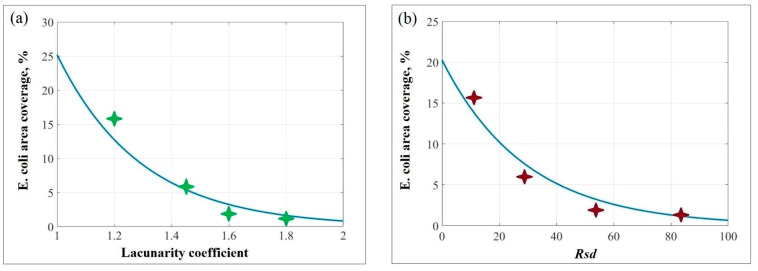
Graphical representation of the E. coli area coverage of biomimetic surfaces as a function of: (**a**) lacunarity coefficient; (**b**) developed interfacial area ratio. The stars on both plots indicate experimental data, while the line indicates the fitted data using second order polynomials.

According to the obtained results, the *E. coli* area coverage decreased rapidly in the following order: flat substrate (18%), magnolia petal replica (16%), pansy petal replica (6%), chamomile petal replica (2%), and rose petal replica (1.5%). It is notable that the surface complexity and heterogeneity of biomimetic substrates increased in the same order (Figure 3). For instance, of all the biomimetic samples, the magnolia petal replica exhibited the lowest degree of surface complexity/heterogeneity (*Sdr* = 12/β = 1.2) and the highest level of the *E. coli* area coverage (16%) which was comparable to the *E. coli* area coverage of the flat substrate (18%). Additionally, similar to the flat substrate, the surface of the magnolia petal replica was heavily covered with bacterial agglomerates and fibrous networks (Figure 4a,b). Despite the presence of periodic microstructures on the magnolia replica surface, they were too flattened and smooth to create insurmountable barriers to bacteria and prevent their network formation. In contrast to magnolia replicas, pansy petal replicas were covered with more convex microstructures which were capable of blocking bacteria between the valleys and limiting their potential for cell-to-cell communications [16]. As a result, reduced *E. coli* area coverage (6%) and significantly fewer bacterial aggregates were observed on the pansy replica surface (Figure 4c). However, the lowest levels of the *E. coli* area coverage (1.5–2%) were exhibited by the rose and chamomile petal replicas. Additionally, no bacterial clusters were observed on their surfaces, and most of the attached bacterial cells were found to be isolated between sub-micron foldings. Considering that chamomile and rose replicas had the highest level of surface complexity and heterogeneity compared with magnolia and pansy ones (Figure 3), the obtained results confirmed the dependence of antibacterial properties of surfaces from their topography parameters, such as the lacunarity coefficient (β) and the developed interfacial area ratio (*Sdr*). Additionally, according to the plotted graphs shown in Figure 5, the *E. coli* area coverage of flower petal replicas decreased exponentially with the increase in β and *Sdr* parameters (Figure 5a and Figure 5b, respectively), and the most pronounced antibacterial properties could be observed at β > 1.6 and *Sdr* > 50. Thus, the obtained results can be used to predict the antibacterial properties of biomimetic surfaces using the fractal analysis of their SEM images.

## 4. Conclusions

In this study, we demonstrated the effectiveness of fractal image analysis of biomimetic silicone surfaces for predicting their antibacterial properties. During this research, we applied a soft lithography technique to replicate the surface of various flower petals, including magnolia, pansy, chamomile, and rose petals. Our comprehensive analysis of surface topography and antibacterial efficiency of the obtained replicas revealed a strong correlation between their antibacterial properties and their surface complexity and heterogeneity. As a measure of surface complexity and heterogeneity, we used the lacunarity coefficient (β) and the developed interfacial area ratio (*Sdr*), which were obtained by a fractal analysis of SEM images representing the replica surfaces. The highest values of β and *Sdr* parameters (*Sdr* = 53–83/β = 1.6–1.8) were observed in chamomile and rose petal replicas whose surfaces were covered with the arrays of hemispherical micro-cavities and sub-micron foldings. These sub-micron foldings align the attached bacteria within the gaps and prevent bacterial clustering. As a result, reduced *E. coli* area coverage (1.5–2%) and inhibited network formation could be observed on the surface of chamomile and rose petal replicas. On the other hand, pansy and magnolia petal replicas with less complex and flattened microstructures on their surfaces showed lower β and *Sdr* values (*Sdr* = 12–29/β = 1.2–1.45) and higher *E. coli* area coverage (9–16%). Our findings indicate that the bacterial area coverage of biomimetic substrates decreased exponentially with increasing β and *Sdr*, and the most pronounced antibacterial properties were observed on biomimetic surfaces with β > 1.6 and *Sdr* > 50. These findings could be used to identify biomimetic materials with superior antibacterial properties through fractal analysis of their SEM images and to produce advanced antibacterial silicone coatings in a cost-effective, time-efficient, and environmentally friendly way.

## Data Availability

The data presented in this study are available on request from the corresponding author.

## Supplementary Materials

The following supporting information can be downloaded at: https://www.mdpi.com/article/10.3390/polym17020213/s1, Table S1: Summary of works on nano- and micro-patterned silicone coatings with antibacterial properties; Figure S1: SEM images of sample sections.
