# Supplementary material for: Biomimetic Silicone Surfaces for Antibacterial Applications
Source: Polymers (Basel). 2025 Jan 16;17(2):213. doi: 10.3390/polym17020213 (PMC11768613; doi:10.3390/polym17020213)
Supplement: Supplementary file 1 [file polymers-17-00213-s001.zip › polymers-3396579-supplementary.pdf]

Supplementary materials

Table S1. Summary of studies on nano- and micro-patterned silicone coatings with antibacterial properties

Manufacturing methods	Shape, height, and width of structures	Bacterial type	Bacterial coverage/ cell destiny	Applications	Ref.*
Electron etching and soft lithography	Nanopillars 220 × 270 nm; 800 × 380 nm	*Escherichia coli*; *Staphylococcus aureus*	1.5%, 2.0% 10.0%, 27.0%	Antibacterial coatings	[19]
Electron etching and soft lithography	Nanopillars 400 × 500 nm; 400 × 700 nm	*Escherichia coli*	3.0%, 10.0%	Antibacterial coatings	[20]
Soft lithography	Square and circular pillars/cavities/ridges 115 nm × 3–6 µm	*Staphylococcus epidermidis*; *Bacillus subtilis*; *Escherichia coli*	0.8-2.0 * 10^6/cm^2 0.1-0.3 * 10^6/cm^2	Antibacterial coatings for biosensors/ bioelectronics	[21]
Soft lithography (sandpaper) and iCVD	Arbitrary-shaped microstructures 8 µm; 36 µm	*Escherichia coli*; *Staphylococcus aureus*	7-log reduction 3-log reduction	Antibacterial coatings	[22]
Laser machining	Sharklet, circular/square pillars/ridges/channels 10–20 × 20–90 µm	*Escherichia coli*	5-10 times reduction	Antibacterial coatings for medical/ domestic use	[23]
Laser machining and soft lithography	Conical microstructures 1–9 × 20–40 µm	*Escherichia coli*; *Staphylococcus epidermidis*	2.0-80.0 %	Antibacterial coatings	[24]
Biomimetic soft lithography (dried shark skin)	Placoid scale shaped microstructures 150–300 µm	*Escherichia coli*; *Staphylococcus hominis*	2.0%	Wound dressings	[25]
Biomimetic soft lithography (*Laminaria japonica*) + poly(GHPEI) coating	Mound-shaped microstructures 500 nm × 8 µm	*Escherichia coli*	3.8%	Antibacterial coatings	[26]

* The reference numbers correspond to the references in the main text.

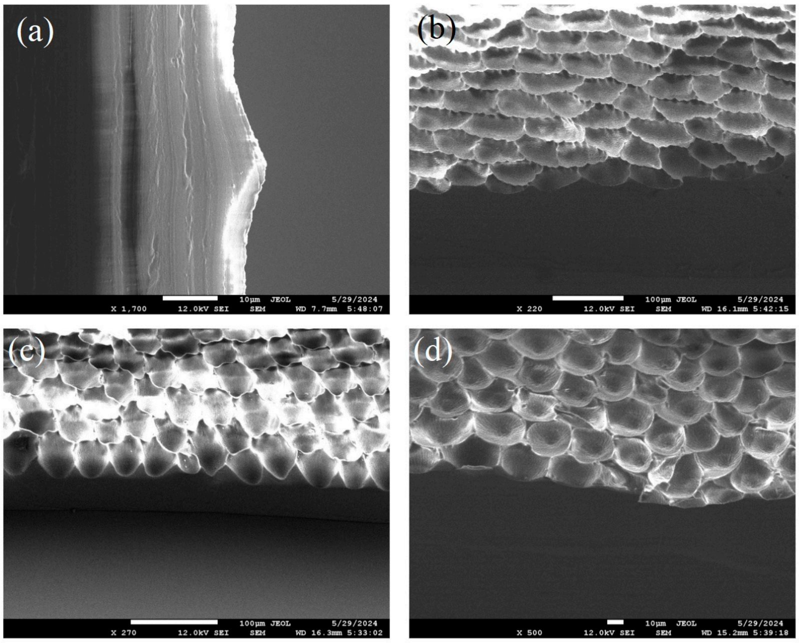

Fig. S1. SEM images of sample sections: *a) magnolia replica sample at an angle of 90°; b) pansy replica sample at an angle of 45°; c) chamomile replica sample at an angle of 45°; d) rose replica sample at an angle of 45°*
